# Evans Blue as a Simple Method to Discriminate Mosquitoes’ Feeding Choice on Small Laboratory Animals

**DOI:** 10.1371/journal.pone.0110551

**Published:** 2014-10-21

**Authors:** Ceres Maciel, André Fujita, Daniele I. Gueroni, Anderson D. Ramos, Margareth L. Capurro, Anderson Sá-Nunes

**Affiliations:** 1 Laboratório de Imunologia Experimental, Departamento de Imunologia, Instituto de Ciências Biomédicas, Universidade de São Paulo, São Paulo, São Paulo, Brasil; 2 Departamento de Ciência da Computação, Instituto de Matemática e Estatística, Universidade de São Paulo, São Paulo, São Paulo, Brasil; 3 Laboratório de Mosquitos Geneticamente Modificados, Departamento de Parasitologia, Instituto de Ciências Biomédicas, Universidade de São Paulo, São Paulo, São Paulo, Brasil; 4 Instituto Nacional de Ciência e Tecnologia em Entomologia Molecular (INCT-EM), Rio de Janeiro, Rio de Janeiro, Brasil; University of Cincinnati, United States of America

## Abstract

**Background:**

Temperature, humidity, vision, and particularly odor, are external cues that play essential roles to mosquito blood feeding and oviposition. Entomological and behavioral studies employ well-established methods to evaluate mosquito attraction or repellency and to identify the source of the blood meal. Despite the efficacy of such methods, the costs involved in the production or acquisition of all parts, components and the chemical reagents involved are unaffordable for most researchers from poor countries. Thus, a simple and relatively low-cost method capable of evaluating mosquito preferences and the blood volume ingested is desirable.

**Principal Findings:**

By using Evans blue (EB) vital dye and few standard laboratory supplies, we developed and validated a system capable of evaluating mosquito’s choice between two different host sources of blood. EB-injected and PBS-injected mice submitted to a number of situations were placed side by side on the top of a rounded recipient covered with tulle fabric and containing *Aedes aegypti* mosquitoes. Homogenates from engorged mosquitoes clearly revealed the blood source (EB- or PBS-injected host), either visually or spectrometrically. This method was able to estimate the number of engorded mosquitoes, the volume of blood ingested, the efficacy of a commercial repellent and the attractant effects of black color and human sweat.

**Significance:**

Despite the obvious limitations due to its simplicity and to the dependence of a live source of blood, the present method can be used to assess a number of host variables (diet, aging, immunity, etc) and optimized for several aspects of mosquito blood feeding and vector-host interactions. Thus, it is proposed as an alternative to field studies, and it could be used for initial screenings of chemical compound candidates for repellents or attractants, since it replicates natural conditions of exposure to mosquitoes in a laboratory environment.

## Introduction

Several external cues are involved in mosquito behavior and contribute to mating, blood feeding and oviposition. Although temperature, humidity and vision play essential roles in the mosquito’s life cycle, odor seems the most important variable by far for medium-to-long range attraction [1]–[4]. In fact, olfactory signals are essential stimuli for behavioral features and host-seeking by hematophagous mosquitoes. Through their sensitive olfactory organs, mosquitoes are able to choose more attractive hosts over less attractive ones. And finding a suitable host will a have positive impact on reproduction, as blood components are essential for the development of eggs [5]–[7]. Elementary aspects of mosquito olfaction and odorant reception are covered by a vast literature [1], [3], [8]–[11] in studies that include *Aedes aegypti*, *Anopheles gambiae* and *Culex quinquefasciatus* species, the “big three” mosquito vectors of emerging and re-emerging human tropical diseases [12].

A number of studies evaluating putative attractants or repellents to mosquitoes and their odor-mediated orientation responses usually employ commercial or “home-made” olfactometers of variable complexity [13]–[21]. In order to fulfill most external cues needed for studying mosquito behavior, this equipment usually has a flight chamber, tubes and connections, temperature/humidity control, filters, a pump to generate and control airflow, and other devices. Despite their attested effectiveness in evaluating mosquito responses, the cost involved in the production or acquisition of all these components is prohibitive for researchers from poor countries. In addition, given the size and shape of this kind of equipment, special requisites are needed for its storage and transportation.

Blood meal identification is another important aspect to understand host preference and the chemoreceptive nature of mosquito behavior. A number of serological and non-serological procedures have been employed to identify the blood source, the precipitin test being the most widely used by far [22]. Nevertheless, some of these procedures present inconsistent degrees of specificity and sensitivity, and some others are complex and require laboratory reagents that need to be refrigerated, another limiting factor for groups with limited financial resources. On the other hand, the evaluation of mosquito preferences toward two or more known individual hosts seems more attainable, however, a convenient method to assess this information is still lacking. All these things considered, studies of mosquito attractiveness or repellency would benefit from a simpler, spaceless and low-cost method able to discriminate mosquitoes choice toward small vertebrate hosts submitted to different treatments, experiences or situations, placed side by side either in closed or open environments. By using a well-known vital dye, Evans blue (EB), together with standard and affordable laboratory supplies, we standardize and validate here a methodology to evaluate the source of a mosquito’s blood meal when exposed to two alive feeding options under the same conditions.

## Methods

### Mice and mosquitoes

Female BALB/c and C57BL/6 mice, 6–16 weeks old, were bred and maintained at the Department of Immunology, Institute of Biomedical Sciences, University of São Paulo, Brazil. Each set of experiments consisted in 3 to 6 replicates (rounds) comparing 2 mice of similar age and weight placed side by side (meaning 3–6 mice per group). Mice from each round were never reused in another round, as evidences from our group show that mosquito’s feeding behavior in preexposed hosts is altered (data not shown). All the experiments involving laboratory animals were evaluated by the “Ethics Committee for Animal Use” from Institute of Biomedical Sciences - University of São Paulo (our Institutional Animal Care and Use Committee) and approved under the protocol numbers 91/2009 and 140/2011. The procedures are according to the Brazilian National Law number 11794 from 10/08/2008, which regulates all research activities involving animal use in the country.

Male and female *A. aegypti* were reared in an insectary at the Department of Parasitology, Institute of Biomedical Sciences, University of São Paulo, Brazil. Temperature was maintained at 27°±1°C, 75–80% humidity and a 12∶12-h light:dark cycle. Larvae were fed on powdered fish food (Tetramin, Blacksburg, VA, USA) and adult mosquitoes were given continuous access to a 10% sucrose solution that was removed 12 h prior to the blood feeding experiments [23].

### Absorbance spectrum measurement

A mouse was bled through a small cut at the tip of the tail and 5 µL of blood were drawn and diluted to 250 µL (1∶50 dilution) with distilled water to promote cell lysis. This sample was centrifuged (10 min/500 *g*) to sediment particles/cellular debris and the supernatant was collected. Evans blue (EB) solution (Sigma Aldrich, St. Louis, MO, USA) was also prepared in distilled water at 200 µg/mL. Absorbance scanning was performed in blood and EB samples (220 to 720 nm) at room temperature employing a NanoDrop 2000 spectrophotometer (Thermo Fisher Scientific, Wilmington, DE, USA), using distilled water as blank.

For *ex vivo* evaluations, mice were injected i.v. with 0.2 mL of PBS only or EB diluted in PBS (20 and 200 mg/kg) through the tail vein. After 10 min, 5 µL of blood from each mouse were drawn from the same vein, diluted to 250 µL of distilled water (1∶50 dilution) and centrifuged for 10 min/500 *g*. Two-hundred microliters of the supernatant were transferred to a flat bottom 96-well plate (TPP Techno Plastic Products AG, Trasadingen, Switzerland) and absorbance was evaluated at 540 and 620 nm in a microplate reader (SpectraMax 190, Molecular Devices, Sunnyvale, CA, USA).

### Exposure to mosquito bites

Male and female *A. aegypti* mosquitoes were placed inside rounded transparent plastic containers (12 cm diameter) covered with a tulle fabric, in a 1∶2 proportion (25 male to 50 female, approximately). Mosquitoes were kept without sucrose for 12 h prior to the experiments. On each round, one PBS-injected and one EB-injected BALB/c mouse were subcutaneously anesthetized (in the dorsal region to avoid interference with mosquito’s feeding in the ventral region) and placed on the top of the tulle screen, side by side for 30 min, so that mosquitoes had direct contact with the animals’ skin for enough time to acquire their blood meal. After mosquito’s exposure, mice were euthanized and the plastic containers were placed in a freezer for 30 min to kill all mosquitoes.

Dead mosquitoes were individually placed in 1.7 mL microcentrifuge tubes (Corning Life Sciences – Axygen Inc., Union City, CA, USA) containing 250 µL of distilled water and grinded with small plastic disposable pestles (Corning – Axygen) until total homogenization. Samples were centrifuged for 5 min/300 *g* and 200 µL of debris-free supernantants were transferred to a flat bottom 96-well plate. In order to roughly estimate the blood volume ingested by each mosquito, 20 µL of blood from PBS- and Evans blue-injected mice were drawn prior to each experiment and diluted to 500 µL of distilled water. Half of the volume was kept and the other half was serially diluted in distilled water (1∶2 ratio). Two-hundred microliters of each dilution were transferred to the same 96-well plate and absorbance was evaluated at 540 and 620 nm as described above. A linear regression was produced by using optical density values from blood dilutions and used as a “standard curve” to estimate the blood ingested by each mosquito.

### Repellency and attraction studies

To study repellency and attraction, two rectangles measuring 20×50 mm were cut at equal distances in a circular piece of filter paper that was placed on the top of the plastic container, to make sure that the same skin area was exposed to the mosquitoes.

For the repellency studies, one drop of a commercial repellent (OFF!, SC Jonhson Ltda, Manaus, AM, Brazil) was applied in the abdomen of anesthetized BALB/c mice with a cotton swab and let to dry for 30 minutes. In the first set of experiments, the repellent was applied on EB-injected mice. As a counterproof, in the second set of experiments, the repellent was applied on PBS-injected mice. Mice were exposed to female *A. aegypti* mosquitoes and blood feeding was evaluated as previously described. Each set of experiment was carried out in three totally independent biological replicates (for each replicate, a new set of mice, mosquitoes, and equipment was used).

As many studies show that mosquitoes tend to prefer dark colors, we tested whether host color would affect *A. aegypti* feeding behavior. BALB/c (white coat) and C57BL/6 (black coat) mice were placed on the screen side by side for 30 min and blood feeding was evaluated as described above. In one set of experiments, BALB/c mice were injected with EB and C57BL/6 mice were injected with PBS. As a counterproof, in another set of experiments, the BALB/c mice were injected with PBS and C57BL/6 mice were injected with EB. Mice were exposed to female *A. aegypti* mosquitoes and blood feeding was evaluated as previously described. Each set of experiment was carried out in totally independent biological triplicates as explained earlier.

Next, the attractant effect of human secretions was evaluated. The sweat of a human volunteer was collected during intense physical activity in a 50 mL tube, maintained at room temperature, and used not later than 24 hours after collection. Two hundred-microliters of sweat or PBS (used as a negative control) were applied to the abdomen of anesthetized mice with a cotton swab. In the first set of experiments, the sweat was applied on EB-injected mice while PBS was applied to PBS-injected mice. As a counterproof, in the second set of experiments, the sweat was applied on PBS-injected mice while PBS was applied in EB-injected mice. Mice were exposed to female *A. aegypti* mosquitoes and blood feeding was evaluated as previously described. Each set of experiment was carried out in totally independent biological triplicates as explained earlier.

### Statistical analysis

Since all biological replicates were conducted with different sets of mice, mosquitoes, and equipment, we considered the replicates as independent and combined the dataset into one for each experiment. Equality between medians in blood intake was performed using Wilcoxon test. To verify whether two mice are equally chosen by mosquitoes in the different experiments a binomial test was carried out. A *p-value*≤0.05 was considered as statistically significant.

## Results

### Differential blood and EB spectroscopic profiles

**Figure 1:**
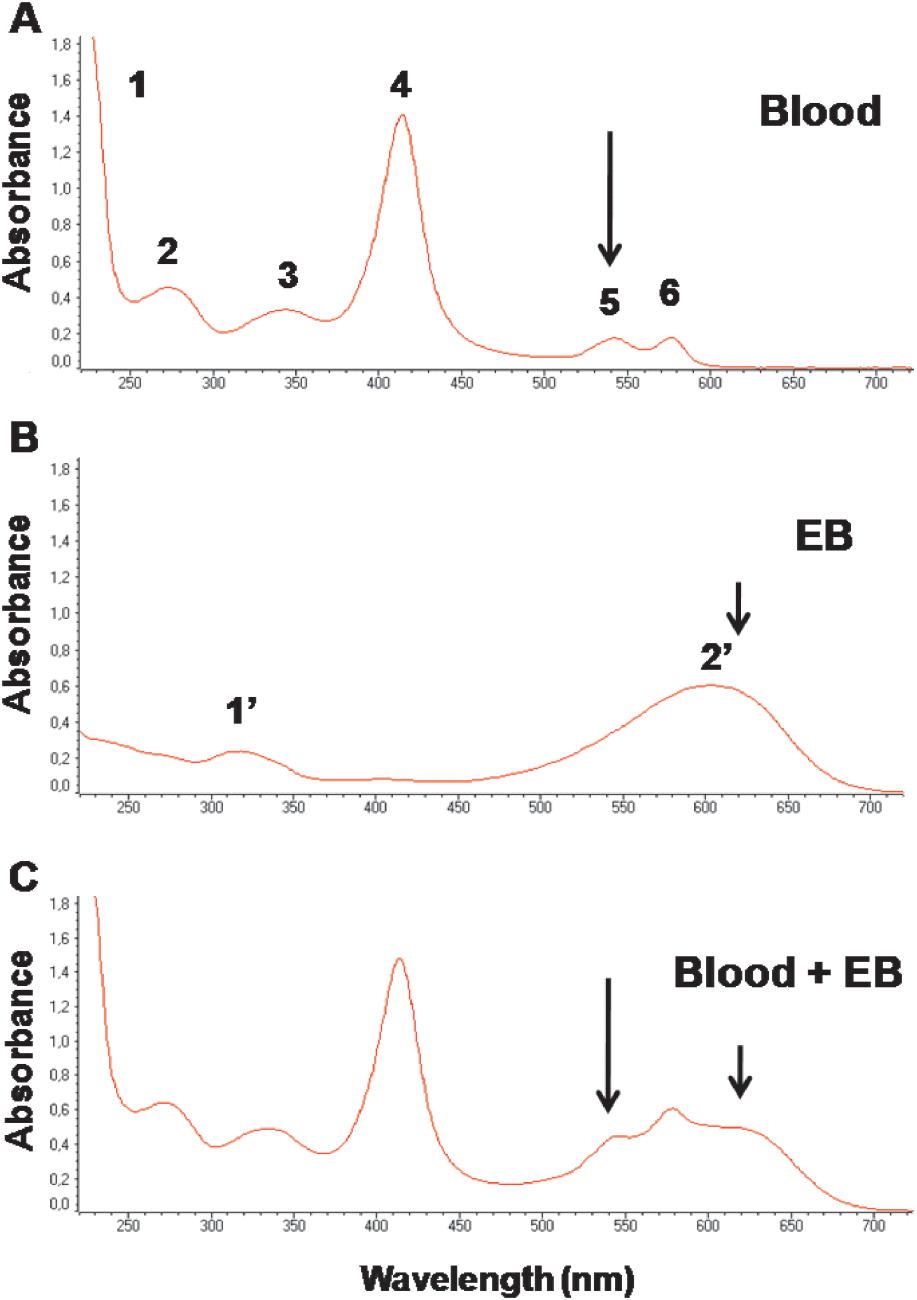
UV-Visible absorption spectra of blood and EB diluted in water. *A.* Five microliters of a BALB/c mouse blood were drawn and diluted to 250 µL (1∶50 dilution) with distilled water, centrifuged and the supernatant collected for analysis. Indicated peaks are: 1) protein amide backbone and nucleic acids (220 nm); 2) proteins with chromophoric amino acids and other small chromophoric molecules (280 nm); 3) globin-heme interaction (340 nm); 4) soret band (420 nm); 5) oxyhemoglobin *β*-band (∼540 nm); and 6) oxyhemoglobin α-band (∼575 nm). *B.* Evans blue (EB) solution was prepared in distilled water at 200 µg/mL. Indicated peaks are: 1′) unkown (∼320 nm); 2′) EB (∼620 nm). *C.* Mixture of A and B solutions v/v. Arrows show the common wavelength to measure hemoglobin (540 nm) and EB (620 nm).

In order to evaluate the spectroscopic profile of blood and EB diluted in distilled water, we determined the absorption spectra of the samples. Figure 1A shows that the blood sample presented at least 6 peaks of absorbance at different wavelengths ranging from 220 to 600 nm. Based on the literature regarding multiwavelenght ultraviolet-visible transmission spectroscopy evaluations of blood and plasma, the peaks represent: 1) protein amide backbone and nucleic acids (220 nm); 2) proteins with chromophoric amino acids and other small chromophoric molecules (280 nm); 3) globin-heme interaction (340 nm); 4) soret band (420 nm); 5) oxyhemoglobin *β*-band (∼540 nm); and 6) oxyhemoglobin α-band (∼575 nm) [24], [25]. On the other hand, Figure 1B shows that the EB diluted in water presents a much simpler absorption spectrum, with two maxima: 1′) at 310–330 nm; and 2′) at 600–620 nm, as previously reported [26]. As expected, mixing EB and blood solutions at 1∶1 ratio, generates an additive spectrum with 7 peaks, being the blood peaks number 5 and 6 and the EB peak number 2′ blended (Figure 1C).

### 540 and 620 nm absorbances discriminate blood samples from PBS- and EB-injected mice

**Figure 2:**
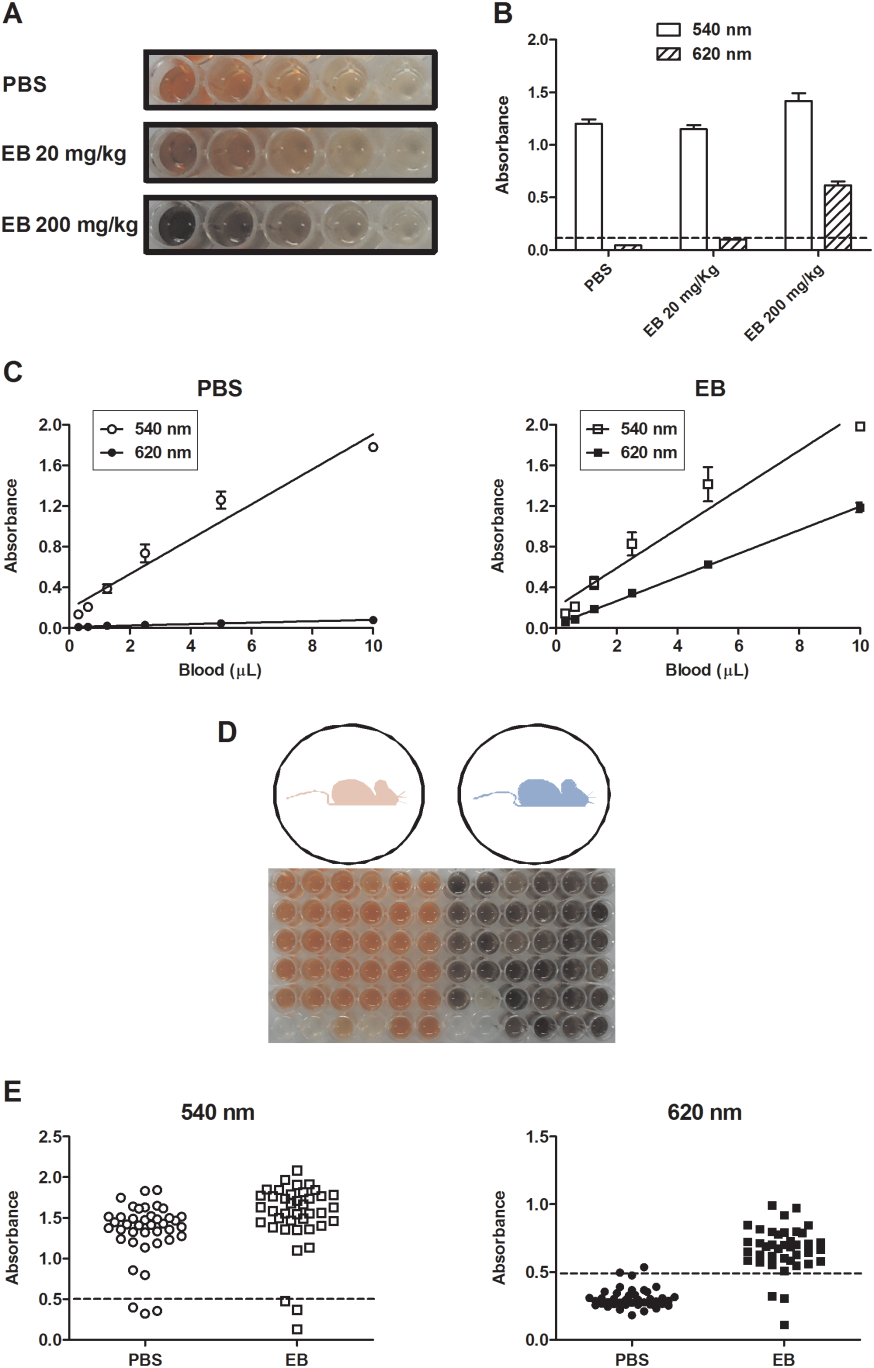
Presence of EB is detected either visually or spectrophotometrically in blood samples or in homogenates of engorged mosquitoes. *A.* Visual comparison of blood dilutions from PBS- and EB-injected mice (20 and 200 mg/kg). *B.* Absorbance comparison of blood from PBS- and EB-injected mice at 540 and 620 nm. *C.* Linear regression of blood volume (X axis) *versus* absorbance (Y axis) at 540 nm and 620 nm from PBS- and EB-injected mice. *D.* Experimental scheme and typical visual profile of homogenates from *A. aegypti* mosquitoes fed on PBS-injected (red) or EB-injected (blue) mice. *E.* Absorbance profiles of homogenates from individual *A. aegypti* mosquitoes fed on PBS- or EB-injected mice at 540 nm and 620 nm.

We next determined the intravenous dose of the dye required to produce fully distinguished blood samples in injected mice. Blood samples collected from PBS- and EB-injected mice and appropriately diluted in distilled water (see Material and Methods) were evaluated either visually or by a spectrophotometer. Compared to PBS-injected mice which present a bright red blood after dilution in water, mice injected with 20 mg/kg of EB present a light “brownish” blood color while those injected with 200 mg/kg present a blue/dark brown blood in the same conditions (Figure 2A). Because spectrophotometric hemoglobin estimations are usually performed at 540 nm and EB presence is evaluated at 620 nm, we determined the absorbance of blood diluted in water from PBS- and EB-injected mice at both wavelengths. Thus, we confirmed that 200 mg/kg is the most appropriated EB dose to distinguish the source of blood samples, since readings of blood from groups injected with 20 mg/kg were about the same of those from PBS-injected group (dotted line - Figure 2B). In fact, when serial dilutions of the samples are evaluated at both wavelengths and plotted as a linear regression (absorbance *versus* blood volume), the visual changes are confirmed (Figure 2C). Blood from PBS-injected mice present a linear absorption curve at 540 nm, but negligible absorption at 620 nm while blood from mice injected with 200 mg/kg EB maintains a linear 540 nm absorption and also presents a linear absorption at 620 nm, indicating the presence of the dye (Figure 2C). Since the correlation coefficient of all regressions were highly significant (r≥0.95; *p*≤0.001), we were able to produce standard curves using blood samples of each mouse (absorbance at 540 nm *versus* blood volume) in order to provide a rough estimation of the blood intake by each individual mosquito.

We next exposed PBS- and EB-injected mice to female *A. aegypti* mosquitoes in separated containers and, after 30 minutes, prepared a whole body homogenate of each mosquito individually and evaluated blood feeding as described in Material and Methods. Figure 2D shows that almost all mosquitoes fed in a similar way, either quantitatively or qualitatively, on both mice and this information is visually readable. The 540/620 nm absorption relation is also able to discriminate the source of the blood meal in homogenates from mosquitoes fed on each mouse. While all mosquitoes’ homogenates present high readings at 540 nm (indicating hemoglobin levels), only homogenates from mosquitoes fed on EB-injected mice present high readings at 620 nm (indicating the presence of the dye). Because unfed mosquito homogenates presented a basal absorption reading either at 540 nm or 620 nm, it was necessary to set a threshold in each experiment to discriminate engorged mosquitoes from those that did not feed (dotted lines – Figure 2E). Importantly, the results of absorbance and visual inspection matched in typically more than 90% of the cases. Complete match was not possible because weaker shades of brown (EB-blood) and red (PBS-blood) were not fully distinguished by naked eyes.

### EB inoculation does not change mice attractiveness to mosquitoes

**Figure 3:**
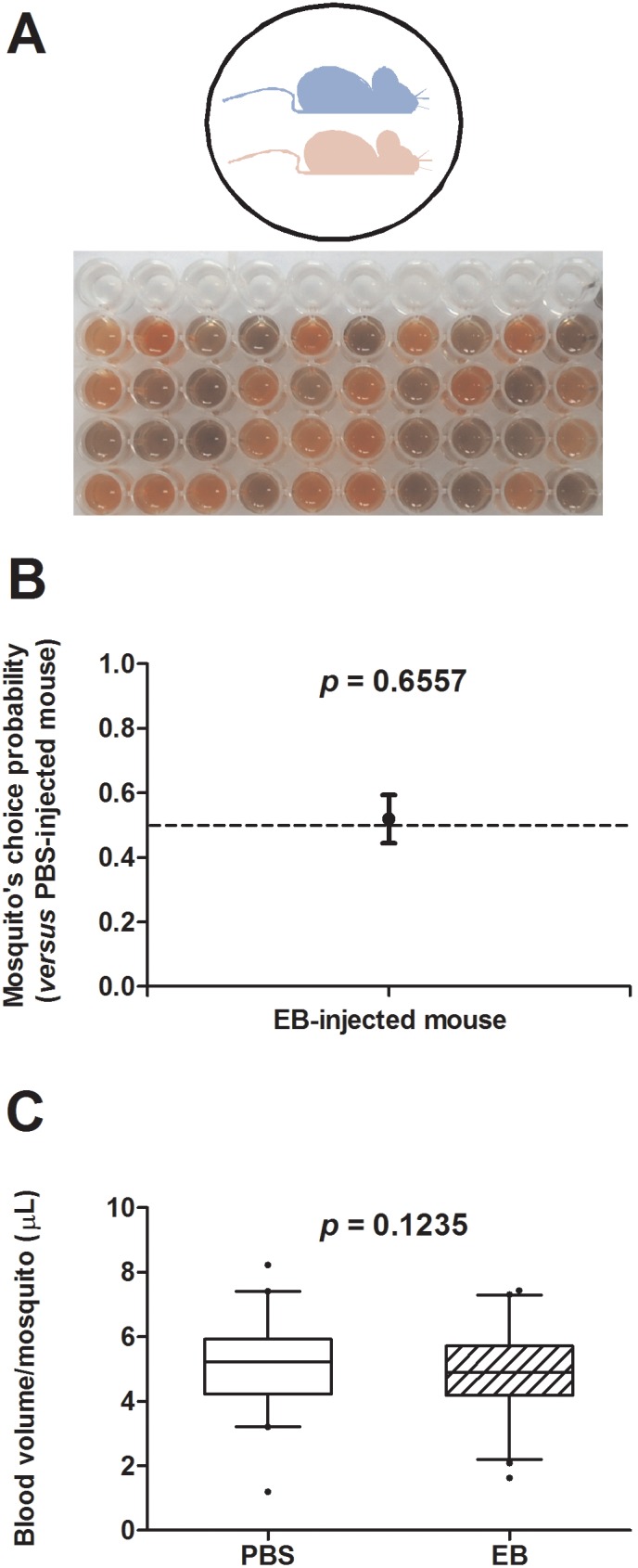
EB presence in blood circulation does not affect *A. aegypti* attractiveness to mice. *A*. Experimental scheme and typical visual profile of homogenates from *A. aegypti* mosquitoes fed on PBS-injected (red) and EB-injected (blue) mice. *B.* Proportion of mosquitoes that chose the EB-injected mouse. The bars represent the 95% confidence interval. *C.* Boxplots of the blood volumes from engorged mosquitoes fed on PBS- or EB-injected mice (2.5–97.5 percentile). Dark dots represent the outliers.

Despite the changes in eye and skin colors between PBS- and EB-injected BALB/c mice, both groups present white coat. In order to investigate if the injection of the dye could somehow interfere with mosquito feeding behavior, we performed a side by side exposure to the same group of mosquitoes, placing both mice (PBS- and EB-injected) on the same container. In these conditions, similar preference was visually observed (Figure 3A), as mosquitoes fed equally (∼52% of the mosquitoes chose EB) on each mouse (*p* = 0.6557 – Figure 3B) and ingested similar amounts of blood (*p* = 0.1235 - Figure 3C). Visual observation also reveals that in our conditions, once a mosquito successfully probes a specific host, it stays on this host until full engorgement, unless it is disturbed (which was not the case in the experiments presented). This assay was independently performed four times and numerical results of individual experiments are presented in the Table S1.

### EB injection model is useful to evaluate mosquito’s attractants and repellents

**Figure 4:**
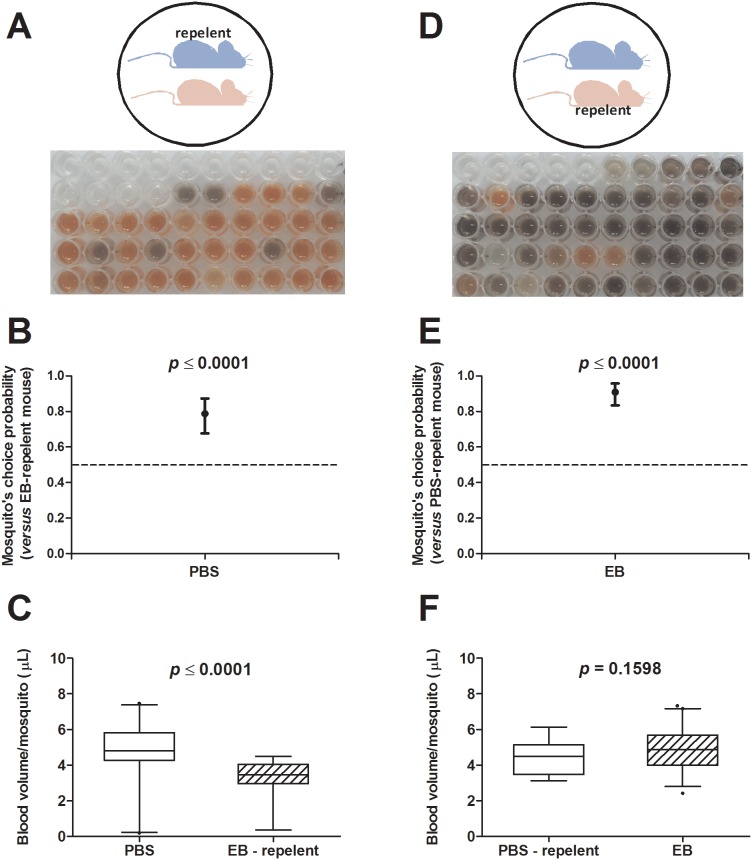
EB model is useful to evaluate the activity of a commercial repellent against *A. aegypti* mosquitoes. *A* and *D*. Experimental scheme and typical visual profile of homogenate from *A. aegypti* mosquitoes fed on PBS-injected (red) and EB-injected (blue) mice that received a commercial repellent before exposure to mosquitoes. *B* and *E*. Proportion of mosquitoes that chose the PBS and EB mice without repellent, respectively. The bars represent the 95% confidence interval. *C* and *F*. Boxplots of the blood volumes from engorged mosquitoes fed on PBS- or EB-injected mice (2.5–97.5 percentile). Dark dots represent the outliers.

We next asked whether our simple and easy system could be employed on attraction and repellency studies. To test the efficacy of a commercial mosquito repellent, one drop of the product was applied on the skin/coat surface of an EB-injected mouse using a cotton swab and paired to a PBS-injected mouse that did not receive any product. Both mice were exposed to mosquitoes and the homogenates prepared as previously described. One representative image shows that the vast majority of the mosquitoes fed on the mouse that had not received the repellent (Figure 4A). The proportion of mosquitoes that chose the mouse without repelent was ∼79% (*p*≤0.0001 - Figure 4B). It is important to point out that the few mosquitoes feeding on EB-repellent group ingested a smaller blood volume than those feeding on PBS group (*p*≤0.0001 – Figure 4C). We repeated the experiment, but now applying the repellent on the PBS-injected mouse and pairing it with an EB-injected mouse that did not receive any product. Again, the majority of mosquitoes (∼91%) fed on the mouse that had not received the repellent (Figure 4D) and this difference was statistically significant as well (*p*≤0.0001 - Figure 4E). However, in this case, there is no statistical evidences to affirm that the blood volume recovered from mosquitoes feeding on the EB group was different to that recovered from the PBS-repellent group (*p* = 0.1598 - Figure 4F), perhaps due to the low number of mosquitoes feeding on the latter group. Each set of experiment was independently repeated three times and the individual data are presented in the Table S2.

**Figure 5:**
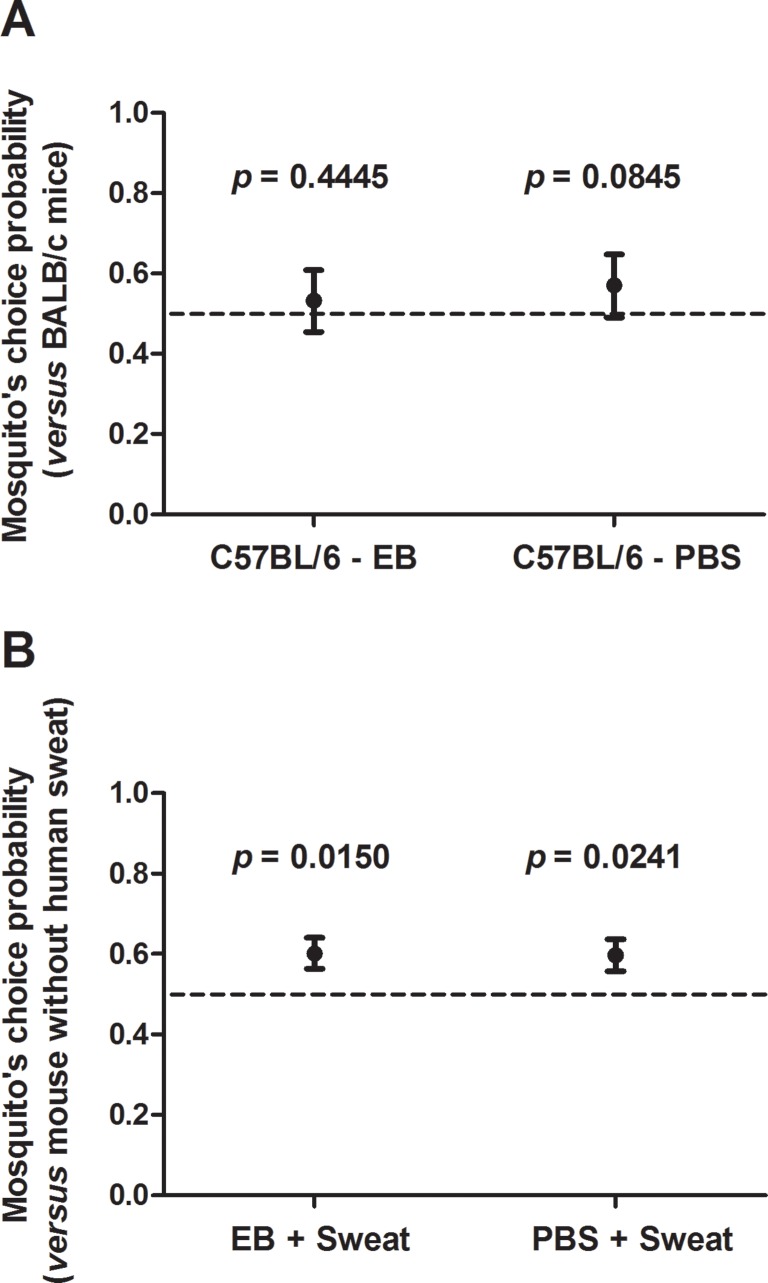
Effect of coat color and fresh human sweat on attraction of *A. aegypti* to mice. *A*. Proportion of mosquitoes that chose the C57BL/6 mouse (black coat). *B.* Proportion of mosquitoes that chose sweat-applied mice (EB-injected or PBS-injected). The bars represent the 95% confidence interval.

Because a number of studies have demonstrated mosquito preference toward black or dark colors than light colors [27]–[29], we evaluated whether the *A. aegypti* mosquitoes from our colony would be more attracted to C57BL/6 mice (black coat) than to BALB/c mice (white coat). Taken together both sets of experiments, our data showed that the proportion of mosquitoes attracted by C57BL/6 mice was ∼53% (when they were injected with EB) and ∼57% (when they were injected with PBS), but there was no statistical evidences to affirm that there is a significant difference in preference (*p* = 0.4445 and *p* = 0.0845, respectively - Figure 5A and Table S3).

In order to test if the volatile chemicals present in human sweat are able to attract *A. aegypti* mosquitoes in our system, we evaluated the number of mosquitoes that fed on mice that received fresh sweat of a volunteer compared to mice that received PBS, always exposing one animal from each group side by side, as described in Material and Methods. The proportion of mosquitoes attracted by human sweat was about the same in both sets of experiments (∼60%) and this preference was statistically significant (*p* = 0.0150 when the sweat was applied on EB-injected mice and *p* = 0.0241 when the sweat was applied on PBS-injected mice - Figure 5B and Table S4). Of note, when the collected sweat was centrifuged and stored at −20°C for many days, its attractant property was lost, perhaps for lacking of volatile compounds and/or skin debris/microbiota (Table S5).

## Discussion

EB is a bis-azo compound originally employed in tissue staining and other biomedical applications by Herbert M. Evans and Werner Schulemann about a century ago [30]. Because of its high affinity for serum albumin, EB was later used to measure blood volume [31] and since then, it has been widely utilized to assess vascular permeability in the blood-brain barrier and other sites and to track lymphatic/plasmatic drainage [32], [33]. Given EB’s relatively low price and broad usage, it was a surprise to us that nobody has tested it as a tool to estimate blood meal volume acquired by hematophagous arthropods. In fact, despite almost 5,300 reports for EB on *PubMed* (as of July 2014), only one work appears when it is crossed with the “mosquito” keyword [34]. However, the mentioned manuscript is not in reality about mosquitoes, but rather it depicts the role of a matrix metalloproteinase in an arbovirus infection into the murine brain. Rare and non-related results are also achieved when EB is crossed with two other relevant groups of arthropod vectors: “sand fly” [35] and “tick” [36]–[38]. A search conducted on the Google Scholar platform unveiled a number of works that used EB to assess the water consumption by mosquitoes and other blood feeding arthropods [39]–[42]. Some other dyes were used to reveal mosquito feeding preferences for sugar solutions [43] and dietary compounds such as amino acids, salts and alkaloids [44]. However, these studies focused on qualitative rather than quantitative parameters and none of them assessed blood-based diets or live sources of blood.

The spectroscopic profile of EB and blood (both diluted in water) revealed that the absorbance peaks are unique of each preparation, except in the 300–350 nm range, and this was confirmed when both solutions were mixed v/v (Figure 1). In order to test if the same profile could be observed *in vivo*, mice were injected with PBS (vehicle) or 2 different concentrations of EB. Figure 2A and 2B confirmed that the blood of mice injected with 200 mg/Kg EB is fully distinguished from the blood of PBS-injected mice, either visually or spectrometrically. Importantly, readings at 540 nm, usually employed to evaluate blood hemoglobin by commercial methods, generates a linear and statistically significant correlation curve (r≥0.95; *p*≤0.001), able to directly estimate blood volumes of up to 10 µL under our conditions for both samples (Figure 2C). On the other hand, readings at 620 nm, routinely used in EB studies, are able to distinguish whether the blood sample comes from PBS- or EB-injected mice (Figure 2C). Again, this distinction is apparent to the naked eyes or through spectrophotometric evaluations (Figure 2D and 2E, respectively). Since unfed mosquito homogenates presented a basal absorption reading either at 540 nm or 620 nm, it was necessary to set a threshold in each experiment to exclude engorged mosquitoes from those that did not feed (Figure 2E - dotted lines). Even though, this simple approach was able to discriminate the host blood source, as long as the mosquito is properly engorged, with a high level of confidence between absorbance readings and visual inspection (matching in more than 90% of cases).

Injection of EB in the circulation turns mice skin “bluish” in color, although no visible change in the animal coat is observed. Because it is not clear to what degree mosquitoes would be able to distinguish between EB-injected and PBS-injected mice, we compared their attractiveness to mosquitoes side by side. None of the parameters evaluated were changed - number of mosquitoes on each mouse or blood volume acquired by each mosquito - when EB- or PBS-injected mice were compared (Figure 3). Although other parameters were not evaluated (e.g.: probing time, biological fitness, etc.), our results indicate that no substantial changes in the mosquitoes’ feeding behavior occurred following EB injection. In addition, the lethal dose-50 (LD_50_) described for EB in mice is 340 mg/Kg [45], and no sign of distress was observed following the 200 mg/Kg injection or during the experiments.

We also tested if our simple method would be efficient enough to evaluate repellency. Another side by side testing was performed using a commercial repellent brand that had its efficacy confirmed, either visually or spectrophotometrically (Figure 4). This assay was carried out as a proof of concept only and for this reason no other parameters associated with repellency were assessed, such as time of protection or number of bites received throughout longer periods of time. Of note, Table S2 shows that a much higher number of non-engorged mosquitoes were recovered from this experiment when compared to those presented in Figure 2, suggesting that repellency affects a small area around the mouse, thus interfering with mosquitoes feeding on the mouse in proximity. In the near future, we intend to further explore this and other potential applications for the system, as the use of spatial repellents has been proposed as an additional strategy for the control of arthropod-borne diseases [46]. In fact, recent reviews focusing on the efficacy of repellents (commercial or from botanical origins) show a number of established compounds and others with great potential [47], [48] as well as different techniques for selecting these repellents [49]. We present our system as an option for an initial screening using live small laboratory animals.

Classic studies from over a century ago have demonstrated mosquito preference toward black or dark colors rather than light colors [27]–[29], [50], although their preference for other colors is less clear. In fact, *Culex quinquefasciatus* mosquitoes preferred black and brown colors under natural light, but did not show any special preference for 4 other colors (white, yellow, blue and skin tone) under the same conditions [51]. On the other hand, *Aedes* spp. were found to be attracted to the following order of colored cloths: black, red, blue, brown, green, white, yellow [28], [52], while *Anopheles maculipennis* preferred red, violet, yellow, and white [53]. Differences were observed even from species of the same genus, as *A. aegypti* were more attracted to darker shades of surface color while *Aedes taeniorhynchus* were attracted to lighter shades [54]. The mosquitoes from our colony, in fact, presented a tendency to prefer C57BL/6 mice (black coat) toward BALB/c mice (white), choosing the former one in 4 out of 6 experiments. However, despite the fact that the number of mosquitoes choosing C57BL/6 was 20% higher than those choosing BALB/c mice, this difference did not reach statistical significance (Figure 5A).

Female mosquitoes are attracted to a number of chemical compounds produced by their hosts, single or in a mixture. The current literature shows that CO_2_ [55], L-lactic acid [13], ammonia [15] and fatty acids [56] are the most effective attractant signals for female *A. aegypti*. These so-called semiochemicals, most of them present in human skin and human sweat [57], are detected by chemosensory receptors distributed across the mosquito body (antennae, mouthparts, wings, and legs) [58]–[60]. Indeed, despite individual degrees of host attractiveness to *A. aegypti* [13], [61], [62], we confirmed that mice receiving fresh sweat from a human volunteer are more attractive to the mosquitoes than those mice receiving PBS only (Figure 5B), corroborating that human sweat has a distinctive “smell/scent” detected by mosquitoes [57]. All results taken together, our method was efficient to identify both repellent and attractive situations and substances to *A. aegypti* mosquitoes and, perhaps, other hematophagous insects.

**Figure 6:**
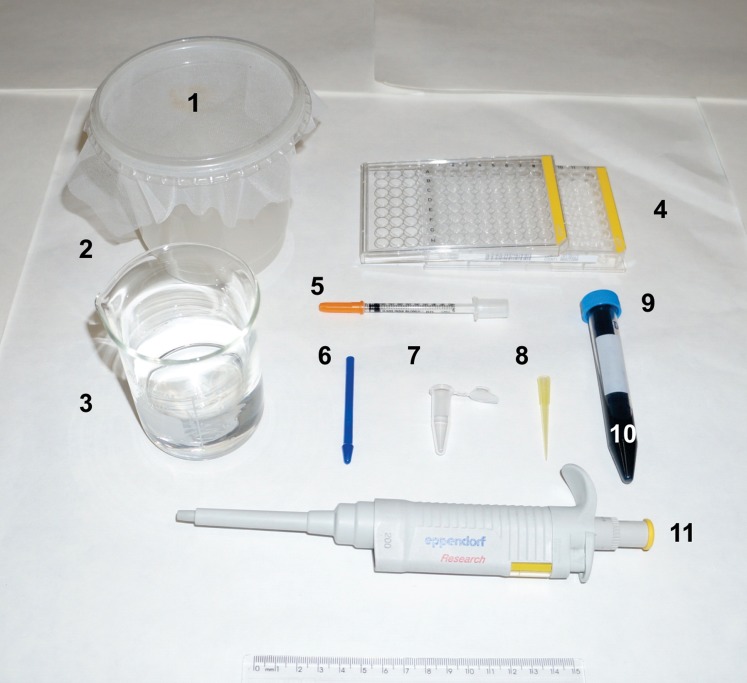
Basic laboratory reagents and supplies needed to evaluate/discriminate the blood source from engorged mosquitoes. Materials are: *1.* Tulle fabric; *2.* Rounded container; *3.* Glass beaker containing distilled water; *4.* 96-well plate, flat bottom; *5.* 1 mL syringe with needle; *6.* plastic pestle; *7.* 1.7 mL microcentrifuge tube; *8.* 20–200 µL universal tip; *9.* 15 mL tube; *10.* EB solution; *11.* 20–200 µL pipette.

Finally, it is important to highlight the limitations and advantages of the method described here. Because of its simplicity, the method is not able to replace olfactometers, since it does not consider a number of variables that may influence mosquito attractiveness. It also relies on live blood sources, and maintaining facilities to breed, feed and raise these animals is costly. Nevertheless, despite the approval of our IACUC, and the use of an EB dose below DL_50_, the ethical limits of animal use on research has been challenged in several countries. In addition, it is not a useful procedure for blood meal identification because it works only with known host species. On the other hand, the method can be validated in the future to alternative sources of blood (e.g.: artificial feeders) and to evaluate whether host cellular and humoral blood factors interfere with mosquito engorgement. Other advantages include: I) the assembly and the small space required to set it up, making the system easy to transport and use virtually anywhere; II) use of conventional laboratory supplies that can be washed and reused while maintaining their performance, as observed in Figure 6; III) even in the absence of a spectrophotometer, the technique is robust enough to be visually evaluated, as evidenced in the Figures 2D, 3A, 4A and 4D; IV) since the method was standardized and validated for live animals, several host parameters can be manipulated (diet, aging, immunity, etc) or added (attractants, repellents, etc). In fact, some of these parameters will be evaluated in our future works, including the role of isolated salivary anti-hemostatic and immunomodulatory molecules on mosquito feeding [63]–[65]; V) the system can be tested for other purposes or optimized to compare more parameters by using alternative vital dyes [66]. Thus, considering all these factors, we believe that a simple and relatively cheap system that requires a small space to be operated would be of interest to entomologists and chemical ecologists and it represents an important contribution to those groups interested in aspects of mosquitoes’ blood feeding and vector-host interactions.

## Supporting Information

Table S1 **Individual data of experiments evaluating EB influence on mice attractiveness to** ***A. aegypti*** **mosquitoes^a^.**
(DOCX)

Table S2 **Individual data of experiments evaluating the effect of a commercial repellent on mice attractiveness to** ***A. aegypti*** **mosquitoes^a^.**
(DOCX)

Table S3 **Effect of color coat on attraction of** ***A. aegypti*** **to mice^a^.**
(DOCX)

Table S4 **Effect of human fresh sweat on attraction of** ***A. aegypti*** **to mice^a^.**
(DOCX)

Table S5 **Individual data of experiments evaluating the effect of human sweat (centrifuged and stored at −20°C for many days) on mice attractiveness to** ***A. aegypti*** **mosquitoes^a^.**
(DOCX)
