# Supplementary material for: Evans Blue as a Simple Method to Discriminate Mosquitoes’ Feeding Choice on Small Laboratory Animals
Source: PLoS One. 2014 Oct 21;9(10):e110551. doi: 10.1371/journal.pone.0110551 (PMC4204902; doi:10.1371/journal.pone.0110551)
Supplement: Table S1 — Individual data of experiments evaluating EB influence on mice attractiveness to A. aegypti mosquitoesa. (DOCX) [file pone.0110551.s001.docx]

**Table S1. Individual data of experiments evaluating EB influence on mice attractiveness to *A. aegypti* mosquitoes^a^**

|  | **Number of mosquitoes** | **Blood volume (μL)**  **Mean ± S.E.M.** |
| --- | --- | --- |
| **Experiment 1 (n= 51)**  PBS  EB  Non-fed/Undetermined | 19  28  4 | 4.696 ± 0.335  4.690 ± 0.195  N.D. |
| **Experiment 2 (n= 50)**  PBS  EB  Non-fed/Undetermined | 23  19  8 | 5.102 ± 0.228  4.626 ± 0.314  N.D. |
| **Experiment 3 (n= 50)**  PBS  EB  Non-fed/Undetermined | 20  20  10 | 5.101 ± 0.253  4.707 ± 0.195  N.D. |
| **Experiment 4 (n= 50)**  PBS  EB  Non-fed/Undetermined | 24  26  0 | 5.597 ± 0.178  5.382 ± 0.204  N.D. |

^a^ On each experiment, one PBS-injected and on EB-injected BALB/c mouse were anesthetized and placed on a tulle screen covering a rounded container with approximately 50 *A. aegypti* female mosquitoes for 30 min. After mosquito’s exposure, the containers were placed in a freezer to kill all mosquitoes and blood feeding was estimated as described in Material and Methods. Consolidated data from these 4 experiments are presented in the Figure 3.

N.D.: not determined.

S.E.M.: standard error of the mean.
