# Supplementary material for: Evans Blue as a Simple Method to Discriminate Mosquitoes’ Feeding Choice on Small Laboratory Animals
Source: PLoS One. 2014 Oct 21;9(10):e110551. doi: 10.1371/journal.pone.0110551 (PMC4204902; doi:10.1371/journal.pone.0110551)
Supplement: Table S2 — Individual data of experiments evaluating the effect of a commercial repellent on mice attractiveness to A. aegypti mosquitoesa. (DOCX) [file pone.0110551.s002.docx]

**Table S2. Individual data of experiments evaluating the effect of a commercial repellent on mice attractiveness to *A. aegypti* mosquitoes^a^**

|  | **Number of mosquitoes** | **Blood volume (μL)**  **Mean ± S.E.M.** |
| --- | --- | --- |
| **Experiment 1 (n= 51)**  PBS  EB-repelent  Non-fed/Undetermined | 26  7  18 | 4.890 ± 0.322  3.231 ± 0.244  N.D. |
| **Experiment 2 (n= 51)**  PBS  EB-repelent  Non-fed/Undetermined | 15  3  33 | 4.305 ± 0.372  2.792 ± 1.246  N.D. |
| **Experiment 3 (n= 52)**  PBS  EB-repelent  Non-fed/Undetermined | 18  6  28 | 4.990 ± 0.316  3.709 ± 0.220  N.D. |
| **Experiment 4 (n= 50)**  PBS-repelent  EB  Non-fed/Undetermined | 1  23  26 | 3.548 ± 0.000  4.680 ± 0.218  N.D. |
| **Experiment 5 (n= 51)**  PBS-repelent  EB  Non-fed/Undetermined | 3  40  8 | 4.186 ± 0.538  4.898 ± 0.177  N.D. |
| **Experiment 6 (n= 51)**  PBS-repelent  EB  Non-fed/Undetermined | 5  27  19 | 4.628 ± 0.518  5.019 ± 0.232  N.D. |

^a^ On each experiment, one PBS-injected and on EB-injected BALB/c mouse were anesthetized and placed on a tulle screen covering a rounded container with approximately 50 *A. aegypti* female mosquitoes for 30 min. After mosquito’s exposure, the containers were placed in a freezer to kill all mosquitoes and blood feeding was estimated as described in Material and Methods. Consolidated data from these 6 experiments are presented in the Figure 4.

N.D.: not determined.

S.E.M.: standard error of the mean
