# Supplementary material for: Evans Blue as a Simple Method to Discriminate Mosquitoes’ Feeding Choice on Small Laboratory Animals
Source: PLoS One. 2014 Oct 21;9(10):e110551. doi: 10.1371/journal.pone.0110551 (PMC4204902; doi:10.1371/journal.pone.0110551)
Supplement: Table S3 — Effect of color coat on attraction of A. aegypti to micea. (DOCX) [file pone.0110551.s003.docx]

**Table S3. Effect of color coat on attraction of *A. aegypti* to mice^a^**

| **Experiment** | **BALB/c** | **C57BL/6** | **C57BL/6 *versus* BALB/c** |
| --- | --- | --- | --- |
| 1 | 31 | 29 | -6.45% |
| 2 | 25 | 31 | +24,00% |
| 3 | 24 | 31 | +29,17% |
| 4 | 17 | 33 | +94.12% |
| 5 | 32 | 22 | -68.75% |
| 6 | 21 | 38 | +80.95% |

^a^ BALB/c (white coat) and C57BL/6 mice (black coat) were anesthetized and placed on a tulle screen covering a rounded container with approximately 50 *A. aegypti* female mosquitoes for 30 min. After mosquito’s exposure, the containers were placed in a freezer to kill all mosquitoes and blood feeding was estimated as described in Material and Methods. Experiments 1, 2 and 3: BALB/c injected with PBS and C57BL/6 injected with EB; Experiments 4, 5 and 6: BALB/c injected with EB and C57BL/6 injected with PBS.
