# Supplementary material for: Evans Blue as a Simple Method to Discriminate Mosquitoes’ Feeding Choice on Small Laboratory Animals
Source: PLoS One. 2014 Oct 21;9(10):e110551. doi: 10.1371/journal.pone.0110551 (PMC4204902; doi:10.1371/journal.pone.0110551)
Supplement: Table S4 — Effect of human fresh sweat on attraction of A. aegypti to micea. (DOCX) [file pone.0110551.s004.docx]

**Table S4. Effect of human fresh sweat on attraction of *A. aegypti* to mice^a^**

| **Experiment** | **PBS** | **Sweat** | **Sweat *versus* PBS** |
| --- | --- | --- | --- |
| 1 | 16 | 31 | +93.75% |
| 2 | 22 | 30 | +36.36% |
| 3 | 23 | 31 | +34.78% |
| 4 | 25 | 25 | 0.00% |
| 5 | 17 | 29 | +70.59% |
| 6 | 16 | 32 | +100% |

^a^ Anesthetized BALB/c mice received two-hundred microliters of PBS or human fresh sweat in the abdomen. Mice were placed on a tulle screen covering a rounded container with approximately 50 *A. aegypti* female mosquitoes for 30 min. After mosquito’s exposure, the containers were placed in a freezer to kill all mosquitoes and blood feeding was estimated as described in Material and Methods. Experiments 1, 2 and 3: PBS group injected with PBS and sweat group injected with EB; Experiments 4, 5 and 6: PBS group injected with EB and sweat group injected with PBS.
