# Supplementary material for: Evans Blue as a Simple Method to Discriminate Mosquitoes’ Feeding Choice on Small Laboratory Animals
Source: PLoS One. 2014 Oct 21;9(10):e110551. doi: 10.1371/journal.pone.0110551 (PMC4204902; doi:10.1371/journal.pone.0110551)
Supplement: Table S5 — Individual data of experiments evaluating the effect of human sweat (centrifuged and stored at −20°C for many days) on mice attractiveness to A. aegypti mosquitoesa. (DOCX) [file pone.0110551.s005.docx]

**Table S5. Individual data of experiments evaluating the effect of human sweat (centrifuged and stored at -20^o^ C for many days) on mice attractiveness to *A. aegypti* mosquitoes^a^**

| **Experiment** | **PBS** | **Sweat** | **Sweat *versus* PBS** |
| --- | --- | --- | --- |
| 1 | 19 | 29 | +52.63% |
| 2 | 24 | 21 | -12.50% |
| 3 | 20 | 26 | +30.00% |
| 4 | 20 | 23 | +15.00% |
| 5 | 26 | 21 | -19.23% |
| 6 | 25 | 11 | -56.00% |

^a^ Anesthetized BALB/c mice received two-hundred microliters of PBS or human sweat in the abdomen. Mice were placed on a tulle screen covering a rounded container with approximately 50 *A. aegypti* female mosquitoes for 30 min. After mosquito’s exposure, the containers were placed in a freezer to kill all mosquitoes and blood feeding was estimated as described in Material and Methods. Experiments 1, 2 and 3: PBS group injected with PBS and sweat group injected with EB; Experiments 4, 5 and 6: PBS group injected with EB and sweat group injected with PBS.

S.E.M.: standard error of the mean
